# A Complete Genome Sequence of the Wood Stem Endophyte *Bacillus velezensis* BY6 Strain Possessing Plant Growth-Promoting and Antifungal Activities

**DOI:** 10.1155/2021/3904120

**Published:** 2021-01-30

**Authors:** Ping Zhang, Jian Diao, Guangqiang Xie, Ling Ma, Lihai Wang

**Affiliations:** Heilongjiang Provincial Key Laboratory of Forest Sustainable Management and Environmental Microbial Engineering, Northeast Forestry University, Harbin 150040, China

## Abstract

An endophytic bacterium *Bacillus velezensis* BY6 was isolated from the wood stems of healthy *Populus davidiana* × *P. alba* var. *pyramidalis* (PdPap). The BY6 strain can inhibit pathogenic fungus *Alternaria alternate* in PdPap and promote growth of PdPap seedlings. In the present study, we used the Pacific Biosciences long-read sequencing platform, a single-molecule real-time (SMRT) technology for strain BY6, to perform complete genome sequencing. The genome size was 3,898,273 bp, the number of genes was 4,045, and the average GC content was 47.33%. A complete genome of strain BY6 contained 110 secondary metabolite gene clusters. Nine of the secondary metabolite gene clusters exhibited antifungal activity and promoted growth functions primarily involved in the synthesis of surfactin, bacteriocins, accumulated iron ions, and related antibiotics. Gene clusters provide genetic resources for biotechnology and genetic engineering, and enhance understanding of the relationship between microorganisms and plants.

## 1. Introduction

*Populus davidiana* × *P. alba* var. *pyramidalis* (PdPap) is the most commonly planted tree species in China. Its characteristics include rapid growth, resistance to drought, and tolerance to low temperatures. PdPap is a female clone; the catkin can fall off naturally, and it is a model species for studying woody plants [1]. With the continuous expansion of plantations under PdPap and degradation of the environment, incidence of leaf diseases caused by fungi is increasing, adversely affecting the growth of PdPap. Leaf blight is one of the fungal diseases of PdPap caused by *Alternaria alternate* [2]. Plant growth-promoting bacteria (PGPB) are frequently used to control plant leaf diseases. PGPB are a type of bacteria that can move freely and usually colonize the internal tissues of a plant. PGPB do not cause substantial damage to host plants, but they instead establish a harmonious symbiotic relationship with the plants [3]. PGPB can inhibit a variety of plant pathogenic fungi [4, 5], promote plant growth and development [6, 7], enhance plant abiotic stress tolerance [8, 9], and regulate and improve the soil environment [10, 11]. *Bacillus* species of bacteria are essential members of PGPR and have been developed as biological agents in agriculture that are used to control various plant diseases [12].

*Bacillus velezensis* is an important member of the PGPR family. Several studies have revealed that *B. velezensis* as an effective bacterium can produce bacteriocins, bioactive compounds, and biosurfactant proteins [13]. Martínez-Raudales et al. [14] reported that a strain of *B. velezensis* 2A-2B, can significantly inhibit the mycelial growth of pathogenic fungi*Phytophthora capsici*, *Fusarium solani*, *Fusarium oxysporum*, and *Rhizoctonia solani* in pepper, and the lowest inhibition rate was more than 60%. Huang et al. [15] inoculated a strain of *B. velezensis* HYEB5-6, in the leaves of *Euonymus japonicus in vitro* and established that it significantly reduced the disease index of *E. japonicus.* In addition, Meng et al. reported that a strain of *B. velezensis* BAC03, can produce IAA, NH3, and ACC-deaminase and promote the growth of 9 types of crops including beets, carrots, cucumbers, pepper, potatoes, radish, squash, tomatoes, and turnips [16]. However, the biocontrol mechanisms of *B. velezensis* species as PGPR have not been clearly defined to date.

In the present study, we isolated and identified a beneficial strain BY6, that has antifungal effects against *A. alternata* and promotes the growth of PdPap. We used the Pacific Biosciences long-read sequencing platform single-molecule real-time (SMRT) technology for strain BY6 to perform complete genome sequencing and annotation to study the genetic basis and molecular mechanisms associated with biological control of the strain BY6. We identified particular secondary metabolic gene clusters for antimicrobial activity and genes linked to promoting plant growth.

## 2. Materials and Methods

### 2.1. Strain Isolation and Identification

The strain BY6 used in our study was isolated from the wood stems of healthy PdPap trees. A growth cone was used to drill into the wood stems of PdPap at a depth of approximately 30 cm (position was 1 m above the ground). The drilling cone and core puller of the growth cone were presterilized at high temperatures of 121°C for 20 mins. Afterwards, wood chips were quickly placed in a 5 mL sterile centrifuge tube. The wood chip samples were then loaded into a centrifuge tube placed in a water bath maintained at a constant temperature. The temperature of the water bath was increased and maintained at 90°C for 2 hours, taking advantage of the heat resistance of spores to eliminate nontarget bacteria. Wood chips were smeared on the surface of the Luria-Bertani (LB) plate 3 times by the streak culture method after natural cooling. The largest colony was transferred to a 1.5 mL cryovial containing 30% glycerol and stored at -80°C after incubating samples for 4 days at 25°C.

We identified the isolated strain. DNA was extracted using a bacterial DNA kit (Tiangen, Beijing, China). The amplification of 16S rRNA was performed according to the method described by Yamamoto and Harayama [17]. DNA amplification conditions were as follows: predenaturation was conducted at 94°C for 5 minutes, and the following procedures were performed after the first cycle: 94°C denaturation for 45 seconds, 55°C primer annealing for 45 seconds, and 72°C primer extension for 1 minute, 30 cycles. The last cycle was conducted at 72°C and extended again for 10 minutes. Products of the polymerase chain reaction (PCR) were sequenced by Shanghai Biotechnology Co. Ltd., and the sequencing results were compared with sequences from the National Center for Biotechnology Information (NCBI) database. A total of 16S rRNA standard sequences were obtained from the GenBank database, and we subsequently used the neighbor-joining method of the MEGA 7.0 software package to construct a phylogenetic tree [18].

### 2.2. Determination of Antagonistic Properties

The antifungal activity of strain BY6 was tested with reference to the method by Chernin et al. [19]. A 5 mm diameter *A. alternata* cake was placed at the center of potato dextrose agar (PDA) plates followed by inoculation with the strain BY6 on the four sides of *A. alternata* at a distance of 2 cm. Diameters of the fungal colonies were measured after 5 days using the cross method. Mycelial growth inhibition was calculated according to the formula: MGI (%) = [(control colony diameter − treatment colony diameter)/treatment group colony diameter] × 100.

### 2.3. Plant Growth Promotion Activity of Strain BY6 on PdPap

We inoculated the strain BY6 in LB culture medium, and the culture was shaken at 160 rpm at a temperature of 25°C for 5 days. Culture was centrifuged at 120,000 rpm for 5 min at 4°C to remove the supernatant, and the bacterial cells were subsequently diluted with sterile water (OD ≈ 1.8 × 10^8^ CFU/mL). The treatment group was inoculated with 50 mL of bacterial cells per pot of PdPap by root irrigation, and the control group was inoculated with 50 mL of sterile water. PdPap seedlings were separated in advance using stem segments, and then transferred into the half-strength Murashige and Skoog (1/2 MS) macronutrient medium (culturing conditions were 30°C, 16 h light). PdPap seedlings were transplanted into a pot containing a mixture of black soil with a vermiculite ratio of 7 : 3 (*v*/*v*) after growing 3 to 5 leaves. The soil mixture used was autoclaved at 121°C for 2 hours before transplanting the seedlings. The seedlings were cultivated in a greenhouse with the following conditions: 25°C, 16 h light : 8 h dark photoperiod, and 40% humidity. The seedlings were watered on a weekly basis with 50 mL of sterile water throughout the growth period. Thirty days after inoculating PdPap seedlings with BY6 according to the method described by Sanchez-Hernandez [20], we selected the 5th to 7th leaves of plants from top to bottom and measured various physiological parameters including plant weight, leaf length, and number of roots.

### 2.4. Genome Sequencing, Assembly, and Annotation

We used the single-molecule real-time (SMRT) technology on the Pacific Biosciences sequencing platform to sequence the complete genome of strain BY6. The SMRT Link version 5.0.1 [21, 22] variant-calling software module was used for preliminary genome assembly, and arrow software was used to align the initial assembly results. GeneMarkS (version 4.17) software was used to predict protein-coding genes of the newly sequenced genome [22] (http://topaz.gatech.edu/GeneMark/). We compared the protein sequences of the predicted genes to the protein database of Clusters of Orthologous Groups (COGs) (http://www.ncbi.nlm.nih.gov/COG) and obtained the corresponding COG annotation results [23]. BLAST software (blastx/blastp 2.2.24+) was used to compare predicted genes with the database of Kyoto Encyclopedia of Genes and Genomes (KEGG) (https://www.genome.jp/kegg/), and then identified relevant pathways for corresponding genes [24, 25]. Gene ontology annotation of the Blast2GO software (http://www.geneontology.org) was used to analyze BLAST results. The antiSMASH program (version 2.0.2) [26] was used to predict secondary metabolite gene clusters of the genome. Finally, we used the Circos software [27] to visualize the sequenced genome.

## 3. Results

### 3.1. The Isolation and Identification of Strain BY6

**Figure 1:**
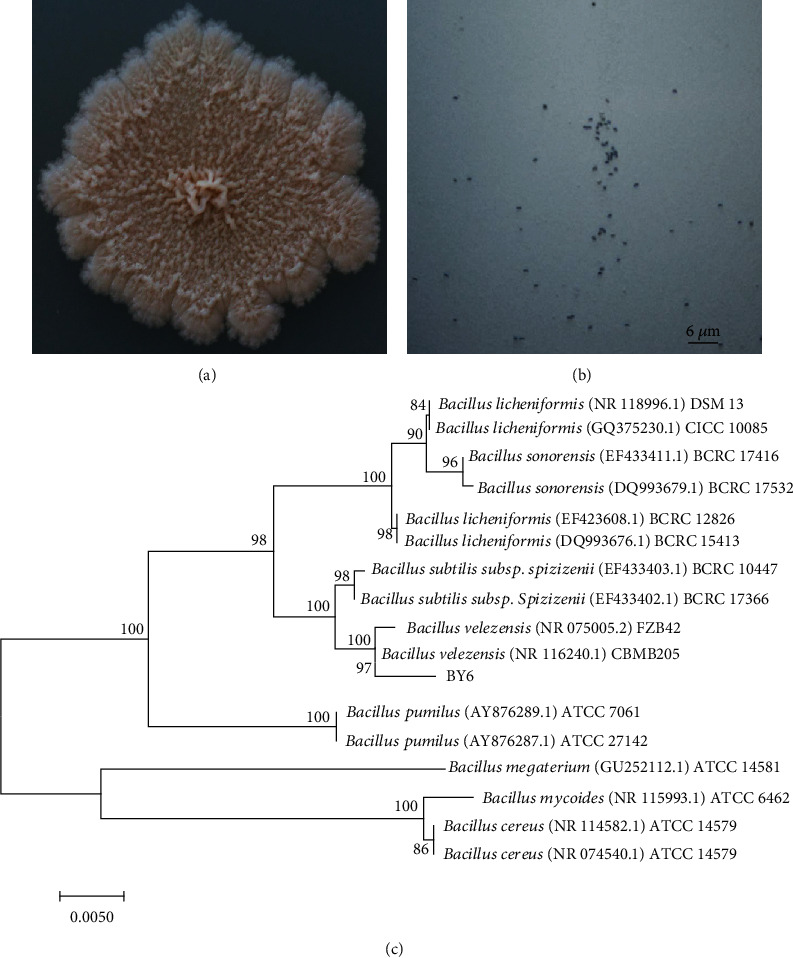
Morphological characteristics and phylogenetic tree of strain BY6. (a) Morphology of the colony. (b) Cell morphology and Gram-staining results. (c). The evolutionary tree constructed by CMEGA 6.0 software and calculation of evolution distance using the adjacency matrices; bootstrap values (expressed as percentages of 1000 replicates) are shown at branch points. The scale bar: 0.005 nucleotide substitution rate.

Strain BY6 was isolated from the wood stems of healthy PdPap seedlings. After culturing BY6 on LB medium at 25°C for 3 days, the colonies were nearly spherical with uneven surfaces, wrinkled, milky white, and odorless (Figure 1(a)). The Gram-staining result was positive, bacterial cells appeared rod-shaped, and spores were formed (Figure 1(b)). MEGA 7.0 software was used to build a phylogenetic tree based on the 16S rRNA gene sequence obtained (Figure 1(c)). Strain BY6 belonged to the same genus branch as *B. velezensis* (NR075005.2) and *B. velezensis* (NR116240.1) exhibiting the closest genetic distance to *B. velezensis* (NR116240.1), with a support rate of 97%. Strain BY6 was identified as *B. velezensis* based on our experimental results.

### 3.2. Inhibitory Efficacy of Strain BY6

**Figure 2:**
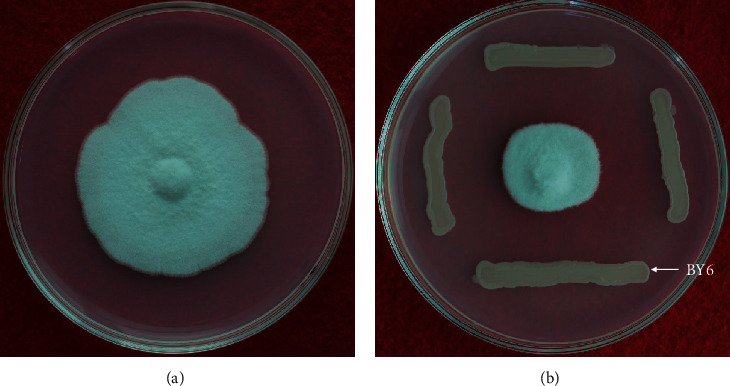
The inhibitory effect of strain BY6 on the growth of *A. alternata* colonies. (a) *A. alternata* colonies cultured separately in PDA medium. (b) Morphology of *A. alternata* colonies and simultaneous inoculation of BY6 in PDA medium. Procedure was repeated four times.

The antifungal activity of strain BY6 against *A. alternata* was tested. After 5 days of coculturing with *A. alternata*, a significant inhibition zone appeared (Figure 2(b)), indicating that strain BY6 inhibited *A. alternata* fungi, the pathogen that causes leaf blight in poplar. A high inhibition rate with a maximum of 64.59% was observed (S 1). In addition, strain BY6 can adapt to extremely cold environments such as isolated areas in the Maoer Mountain (longitude 127°18′0^″^, latitude 45°2′20^″^). It can also withstand high temperatures of 90°C. Consequently, BY6 is a strain of bacteria that can be potentially used as a biocide for controlling plant diseases.

### 3.3. Growth-Promoting Effect of Strain BY6 on Poplar

**Figure 3:**
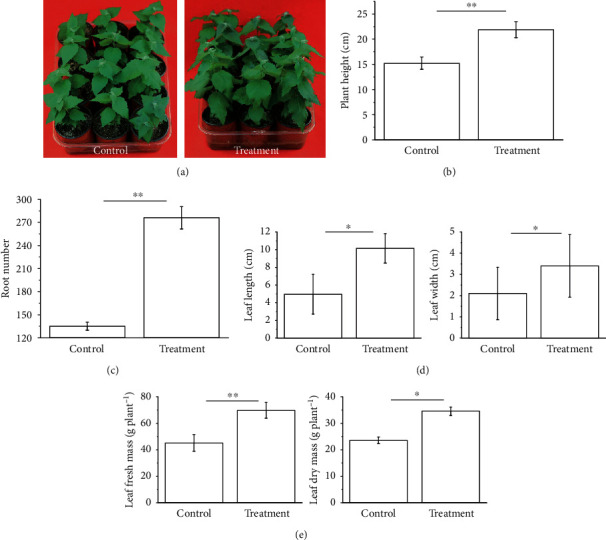
The effect of strain BY6 in promoting growth of PdPap seedlings. (a) Strain BY6 promoted growth of PdPap seedlings under the described soil culture conditions. (b) Heights of PdPap plants. (c) Root numbers of PdPap plants. (d) Length and width of leaves. (e) Fresh and dry weight of leaves. The graphical values are the average of 4 biological repeats. Error bars represent standard errors of the mean (*n* = 4) at each time point; ∗indicates significant difference between treatments,*P* < 0.05, and∗∗indicates a significant difference between treatments,*P* < 0.01.

The growth-promoting effect of strain BY6 on PdPap seedlings was evaluated 30 days after inoculation with strain BY6. The overall growth of PdPap seedlings in the treatment group was significantly higher than that in the control group (Figure 3(a)). Vegetative growth indicators of aboveground and belowground parts of poplar seedlings were assessed (Figures 3(b)–3(e)). Fresh weight, plant height, and root numbers of PdPap seedlings were significantly different between the treatment and control groups (*P* < 0.01). Dry weight, leaf length, and leaf width were significantly different between the treatment and control groups (*P* < 0.05). The results indicate that BY6 has a strong plant growth promotion effect. Therefore, BY6 is a strain that can be potentially used to develop microbial fertilizers to enhance plant growth.

### 3.4. Genome Sequence and Genome Features of Strain BY6

**Figure 4:**
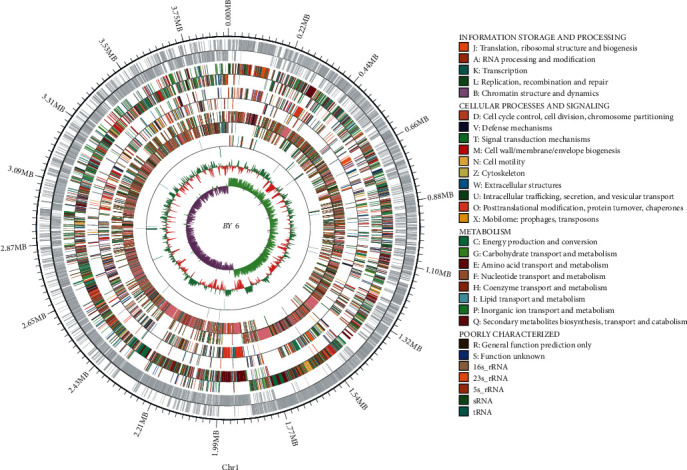
A circular genome map of strain BY6. The outermost circle represents the position coordinates of the genome sequence; from outward to inward are the coding genes, gene function annotation results (including annotation result information for COG, KEGG, and GO databases), ncRNA, and genome GC content, respectively. A calculation of the GC content based on the window (chromosome length/1000) bp and step value (chromosome length/1000) bp. The red section inward indicates that the GC content in that particular region is lower than the average GC content of the whole genome, and the green part outward indicates the reverse. The higher the peak value, the greater the difference between the average GC content and the genome GC skew value. The pink section inward indicates that the content of G is lower than the content of C in that region, and the light green section outward indicates the reverse.

The complete genome for strain BY6 comprised a circular chromosome of 3,898,273 bp with 3,923 protein-coding genes, 27 rRNA genes, 9S rRNA genes, and 86 tRNA genes. The GC content of the chromosome was 47.33%, with 8 gene islands and 16 prophages (Figure 4 and Table 1). The complete sequence of a circular plasmid consisted of 7,256 bp with a GC content of 37.53%; three gene functions are annotated, and they are the tetratricopeptide repeat, the replication protein, and the phenazine biosynthesis-like protein (S 2 and Table 1).

### 3.5. Genetic Basis for Production of Antimicrobial and Plant Growth-Promoting Metabolites in BY6

**Figure 5:**
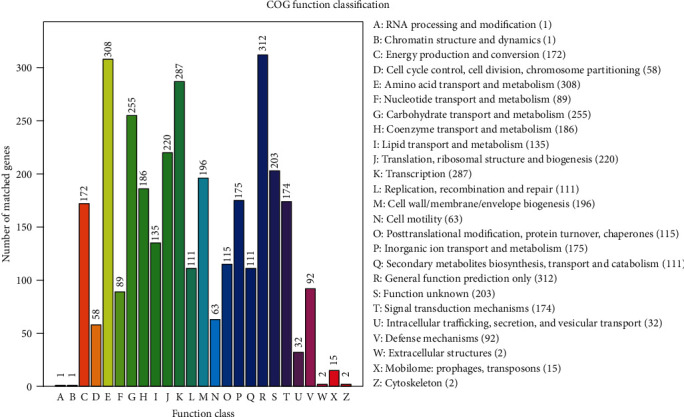
COG functional categories for the complete genome sequences of strain BY6.

In the complete genome sequence of BY6, 3,315 genes were annotated with COG function, accounting for 81.95% of the complete genome sequence. The identified proteins were divided into 25 categories based on their functions (Figure 5). The functions of BY6 proteins were largely amino acid transport and metabolism (9.29%), transcription (8.65%), carbohydrate transport and metabolism (7.69%), translation, ribosomal structure, and biogenesis (6.63%). The numbers of coding genes were 308, 287, 255, and 220. A total of 312 gene proteins were classified as general function prediction only, while the functions of 203 gene proteins were unknown.

We also established that 599 related genes were involved in the bacteriostasis and biosynthesis of secondary metabolites. Analysis of the secondary metabolites of BY6 revealed that there were 9 gene clusters with antibacterial properties (Table 2). Gene clusters were chiefly responsible for anabolic metabolism. The secondary metabolites with potential antibacterial properties included surfactin, bacillibactin, pelgipeptin, bacillolysin, polyketide, bacitracin, macrolactin, and bacillaene, which may be responsible for inhibiting growth of fungal hyphae in *A. alternata*.

A comparison of the BY6 genome sequence with the other three complete genome sequences of this species, LPL-K103 [28], FZB42 [29], and QST713 [30], revealed that the chief gene clusters that produce bacteriostatic secondary metabolites were generally similar. They included biosynthetic gene clusters of surfactin, bacillibactin, pelgipeptin, bacillolysin, polyketide, bacitracin, macrolactin, and bacillaene. However, the biosynthesis gene for sphingosine only occurs in bacterial strain BY6. The strain BY6 has seven other gene clusters associated with secondary metabolism, and their functions are unknown. Moreover, certain enzyme genes linked with antibiotic synthesis such as glucanase, ribonuclease, chitosanase, and peptidoglycanase genes have been characterized in strain BY6.

The BY6 genome contains numerous genes that promote plant growth. We postulated that one secondary metabolic gene cluster produces ferredoxin, a strong iron carrier that increases absorption of iron ions from the soil. BY6 also contains a few other related genes that promote plant growth, including encoding butanone and polyketide among others. Furthermore, BY6 has genes responsible for nitrogen fixation. Therefore, the host plant can absorb these substances to complete its auxin biosynthesis process. Auxin biosynthesis is instrumental in the growth and development of the roots, stems, and leaves of PdPap.

## 4. Discussion

PdPap is one of the most widely planted tree species in China. Various fungal diseases of plant leaves are increasingly occurring with the continuous expansion of PdPap plantations and degradation of the environment, adversely affecting the growth of PdPap. In the present study, the bacterial strain BY6 was isolated from PdPap. Biologically controlled experiments were performed, and results demonstrated that the strain BY6 can inhibit the growth of mycelia of the leaf blight pathogen in PdPap and promote the growth of PdPap seedlings. The endophytic bacterial strain BY6 was isolated from healthy PdPap wood stems. A few studies have indicated that PGPB, which have coevolved with the host plants or are compatible with growth environments, are beneficial to plants. Moreover, PGPB will not cause substantial damage to the host plants, but it can establish a harmonious symbiotic relationship with the host plant [31].

We sequenced the complete genome of bacterial strain BY6 to study its genetic basis and molecular mechanism. We characterized 9 secondary metabolite gene clusters that can inhibit the growth of *A. alternata*. Bacilysin is one of the simplest peptide antibiotics secreted by certain species of *Bacillus* [32]. Bacillaene is a polyene antibacterial substance secreted by *Bacillus* [33]. Surfactin and fengycin are broad-spectrum antibacterial substances present in several *Bacillus* species, and their antibacterial activity has been widely reported [34]. In addition, other genes that can promote plant growth have been identified in strain BY6. There was a similar work on the compounds from *Bacillus amyloliquefaciens* BAS23 that have antifungal activity and plant growth-promoting activity [35]. Genomic information of BY6 will facilitate identification of more active secondary metabolites, which may exhibit strong antibacterial activity against different types of pathogenic bacteria. This is an indication that the strain BY6 has the potential of being used as a biological control agent in the future. Our results further serve as a basis for developing new biocontrol agents or microbial fertilizers using *B. velezensis*.

## Figures and Tables

**Table 1:** Basic characteristics of the complete genome for strain BY6.

Feature	Chromosome	Plasmid
Genome size (bp)	3,898,273	7,256
GC content (%)	47.33	37.53
Protein-coding genes	3,923	0
rRNA genes	27	0
S rRNA genes	9	0
tRNA genes	86	0
Genomic islands	8	0
Prophage	16	0
Total number of genes	4,045	0

**Table 2:** Potential gene clusters encoding secondary metabolites in BY6.

Number	Cluster category	Metabolite Unknown	Position	Function	Reference
1	Nrps	Surfactin Bacillibactin	GM000322-GM000370	Sideropore Antimicrobial	[36, 37]
2	Lantipeptide	Pelgipeptin	GM001260-GM001391	Antimicrobial	[38]
3	Transatpks	Bacillolysin	GM001490-GM001534	Antifungal	[39]
4	Transatpks-nrps	Polyketide	GM001772-GM001824	Antimicrobial	[39]
5	Transatpks-nrps	Bacitracin	GM002910-GM002920	Antimicrobial	[39]
6	Transatpks	Polyketide Macrolactin	GM002309-GM002366	Antibacterial	[28, 40]
7	Bacteriocin-nrps	Bacillaene	GM003051-GM003119	Antibacterial Sideropore	[16, 41]
8	Other	Unknown	GM003682-GM003729		
9	Thiopeptide	Unknown	GM000628-GM000652		
10	Others	Unknown	GM000967-GM001012		
11	Terpene	Unknown	GM001095- GM001120		
12	Terpene	Unknown	GM001998-GM002021		
13	t3pks	Unknown	GM002118-GM002170		

## Data Availability

The available complete genome sequence has been admitted to DDBJ/NCBI/EMBL databases, the received accession numbers is CP051011-CP051012.

## Supplementary Materials

Supplementary 1 Supplementary S1: the effect of strain BY6 on the colony growth inhibition rate of *Alternaria alternata*.

Supplementary 2 Supplementary S2: a circular plasmid map of strain BY6.
