# Supplementary material for: A Complete Genome Sequence of the Wood Stem Endophyte Bacillus velezensis BY6 Strain Possessing Plant Growth-Promoting and Antifungal Activities
Source: Biomed Res Int. 2021 Jan 30;2021:3904120. doi: 10.1155/2021/3904120 (PMC7869414; doi:10.1155/2021/3904120)
Supplement: Supplementary 1 — Supplementary S1: the effect of strain BY6 on the colony growth inhibition rate of Alternaria alternata. [file 3904120.f1.docx]

S 1. The effect of strain BY6 on the colony growth inhibition rate of *Alternaria alternata*.
