# Supplementary material for: A Complete Genome Sequence of the Wood Stem Endophyte Bacillus velezensis BY6 Strain Possessing Plant Growth-Promoting and Antifungal Activities
Source: Biomed Res Int. 2021 Jan 30;2021:3904120. doi: 10.1155/2021/3904120 (PMC7869414; doi:10.1155/2021/3904120)

S 2. A circular plasmid map of strain BY6. From the outward to inward are the functional annotation classification gene (arrow clockwise indicates positive strand coding), Genomic sequence position coordinates, genome GC content. The red section inward indicates that the GC content in that particular region is lower than the average GC content of the whole genome, and the green part outward indicates the reverse. The higher the peak value, the greater the difference between the average GC content and the genome GC skew value. The pink section inward indicates that the content of G is lower than the content of C in that region, and the light green section outward indicates the reverse.


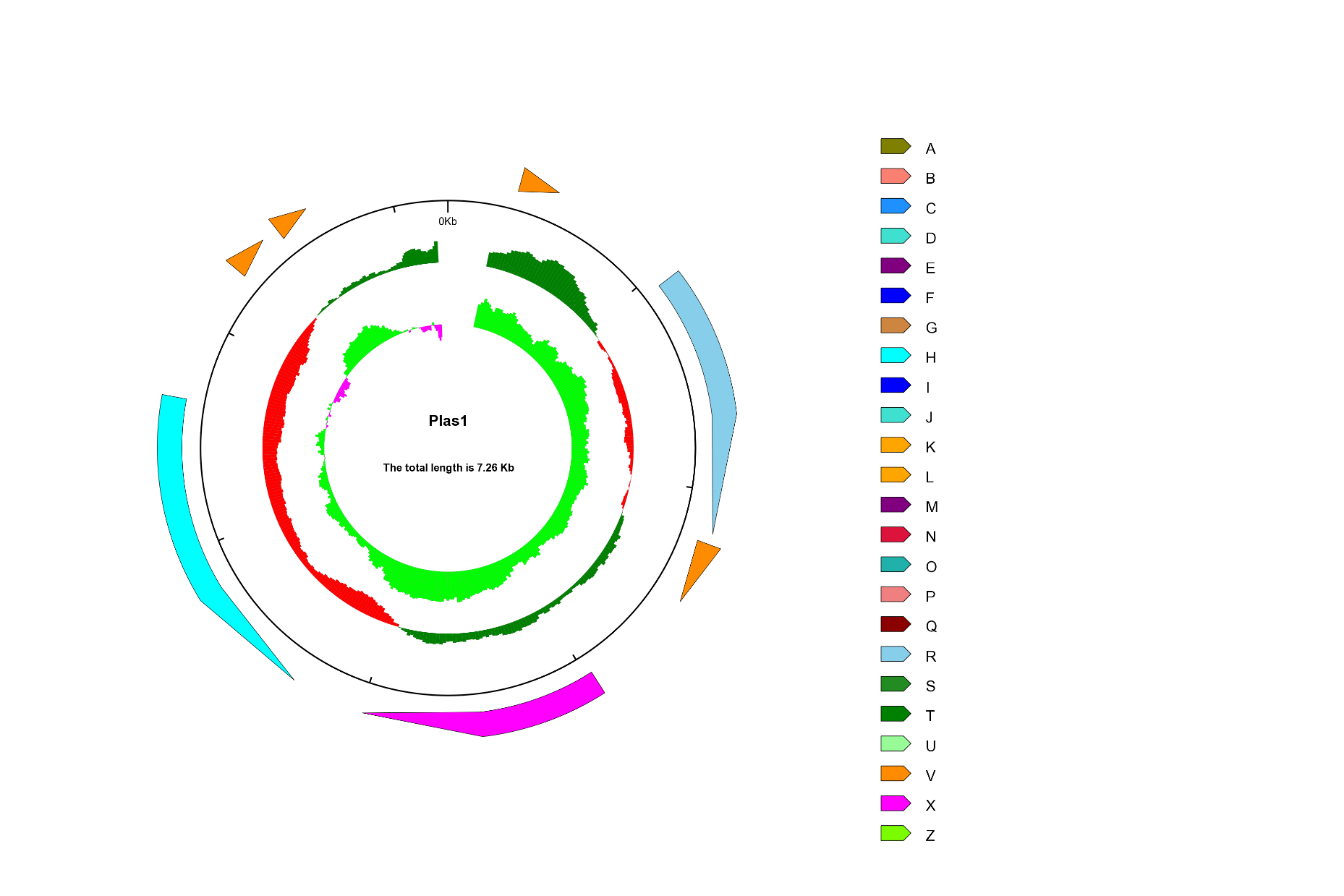

Supplement: Supplementary 2 — Supplementary S2: a circular plasmid map of strain BY6. [file 3904120.f2.docx]
